# Benzyl­ammonium tetra­fluoro­borate 18-crown-6 clathrate

**DOI:** 10.1107/S1600536810021392

**Published:** 2010-06-09

**Authors:** Min Min Zhao

**Affiliations:** a Ordered Matter Science Research Center, College of Chemistry and Chemical, Engineering, Southeast University, Nanjing 211189, People’s Republic of China

## Abstract

The reaction of benzyl­ammonium tetra­fluoro­borate and 18-crown-6 in a methano­lic solution yields the title compound, C_7_H_10_N^+^·BF_4_^−^·C_12_H_24_O_6_O6, which displays a supra­molecular structure. The –NH_3_^+^ substituent of the benzyl­ammonium cation forms a 1:1 supra­molecular rotator–stator structure by N—H⋯O hydrogen-bonding inter­actions.

## Related literature

For similar crown ether clathrates, see: Akutagawa *et al.* (2002 ▶); Kryatova *et al.* (2004 ▶). For their ferroelectric properties, see: Zhang *et al.* (2009 ▶); Ye *et al.* (2009 ▶).
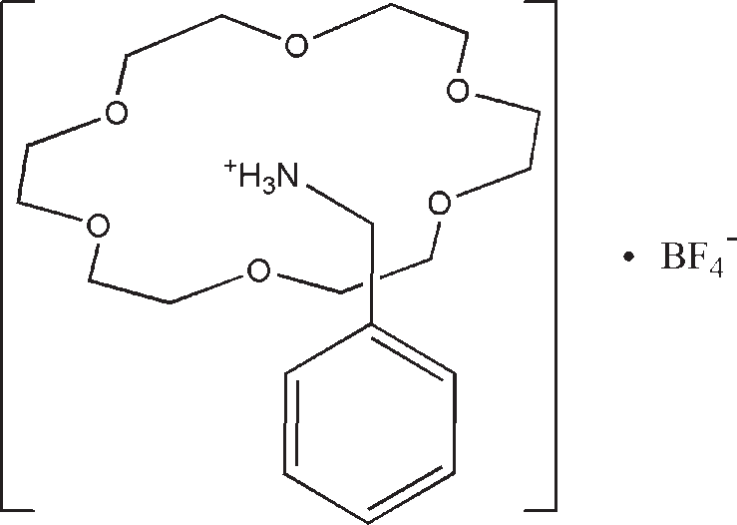


## Experimental

### 

#### Crystal data

C_7_H_10_N^+^·BF_4_^−^·C_12_H_24_O_6_
*M* *_r_* = 459.28
Triclinic, 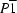

*a* = 9.281 (6) Å
*b* = 10.673 (6) Å
*c* = 11.863 (7) Å
α = 76.418 (16)°
β = 86.244 (17)°
γ = 78.274 (15)°
*V* = 1118.2 (12) Å^3^
*Z* = 2
Mo *K*α radiation
μ = 0.12 mm^−1^
*T* = 293 K
0.40 × 0.30 × 0.20 mm

#### Data collection

Rigaku SCXmini diffractometer
Absorption correction: multi-scan (*CrystalClear*; Rigaku, 2005 ▶) *T*_min_ = 0.958, *T*_max_ = 0.976
12286 measured reflections
5057 independent reflections
4153 reflections with *I* > 2σ(*I*)
*R*_int_ = 0.031

#### Refinement

*R*[*F* ^2^ > 2σ(*F* ^2^)] = 0.045
*wR*(*F* ^2^) = 0.103
*S* = 1.03
5057 reflections
280 parameters
H-atom parameters constrained
Δρ_max_ = 0.25 e Å^−3^
Δρ_min_ = −0.24 e Å^−3^

### 

Data collection: *CrystalClear* (Rigaku, 2005 ▶); cell refinement: *CrystalClear*; data reduction: *CrystalClear*; program(s) used to solve structure: *SHELXS97* (Sheldrick, 2008 ▶); program(s) used to refine structure: *SHELXL97* (Sheldrick, 2008 ▶); molecular graphics: *SHELXTL* (Sheldrick, 2008 ▶); software used to prepare material for publication: *PRPKAPPA* (Ferguson, 1999 ▶).

## Supplementary Material

Crystal structure: contains datablocks I, global. DOI: 10.1107/S1600536810021392/im2207sup1.cif

Structure factors: contains datablocks I. DOI: 10.1107/S1600536810021392/im2207Isup2.hkl

Additional supplementary materials:  crystallographic information; 3D view; checkCIF report

## Figures and Tables

**Table 1:** Hydrogen-bond geometry (Å, °)

*D*—H⋯*A*	*D*—H	H⋯*A*	*D*⋯*A*	*D*—H⋯*A*
N2—H2*A*⋯O6	0.89	1.99	2.866 (2)	167
N2—H2*B*⋯O3	0.89	2.15	2.986 (2)	157
N2—H2*C*⋯O1	0.89	2.05	2.936 (2)	173

**Fig. 1.**
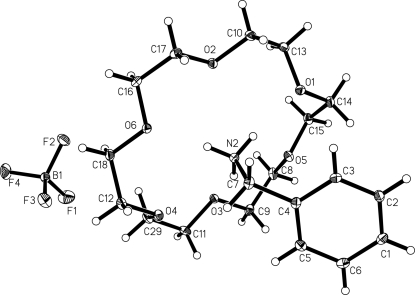
Molecular structure of the title compound showingh the atom numbering scheme. Displacement ellipsoids are drawn at the 30% probability level.

## supplementary crystallographic information

### Comment

There is currently a great deal of interest in crown ethers because of their ability to form non-covalent, H-bonding complexes with ammonium cations both in solid and in solution (Akutagawa *et al.*, 2002; Kryatova *et al.* 2004). Not only the size of the crown ether, but also the nature of the ammonium cation (–NH_4_^+^, RNH_3_^+^, *R*_2_NH_2_^+^, *etc*) can influence both stoichiometry and stability of these host–guest complexes. The host molecules combine with the guest species by intermolecular interactions, and if the host molecule possesses some specific sites, it is easy to realise high selectivity in ion or molecular recognitions. 18-Crown-6 exhibits the highest affinity for ammonium cations RNH_3_^+^, mostly resulting in a 1:1 stoichiometry.

Dielectric permittivity of the title compound was tested to systematically investigate the ferroelectric phase transitions of new materials (Ye *et al.*, 2009; Zhang *et al.*, 2009). The title compound has no dielectric anomaly with the value of 5 and 8 under 1*M* Hz in the temperature range from 80 to 433 K (m.p.> 453 K), suggesting that the compound should show no distinct phase transition occurring within the measured temperature range.

The title compound is composed of cationic [C_7_H_10_N(18-Crown-6)]^+^ and anionic [BF_4_]^–^ ions in a 1:1 stoichiometry (Fig. 1). Supramolecular rotator-like structures are assembled between benzylammonium cations and 18-crown-6 molecules by N—H···O hydrogen-bonding. Intramolecular N—H···O hydrogen bonding lengths are within the usual range around 2.9 Å. No intermolecular hydrogen bond was observed.

The crown ether adopts a conformation in which the rings show some distortion from planarity, with torsion angles: O5—C8—C9—O3 = 64.5 (2) °; O2—C10—C13—O1 = 68.1 (2) °; C16—C17—O2—C10 = 176.0 (1) °; C10—C13—O1—C14 = 179.5 (1) ° and C8—C9—O3—C11 = 167.9 (1) °. C—N bonds of [C_7_H_10_N]^+^ are almost perpendicular to the mean plane formed by oxygen atoms of the crown ether. Boron shows a slightly distorted tetrahedral coordination by four F^–^ ions [range of *cis*-bond angles = 108.9 (1) – 110.2 (1) °; average distance (B—F) = 1.383 (2)–1.397 (2) Å].

### Experimental

C_7_H_10_N^+^BF_4_^–^ (2 mmol, 0.388 g) and 18-crown-6 (2 mmol, 0.528 g) were dissolved in 15 ml methanol. The resulting precipitate was filtered. Two days later, single crystals suitable for X-ray diffraction analysis were obtained from slow evaporation of the remaining methanolic solution at 0°C (yield: 95%).

### Refinement

All hydrogen atoms were calculated geometrically with C—H distances ranging from 0.93 to 0.97 Å and N—H = 0.90 Å. refinement of hydrogen atoms was performed using a riding model with *U*_iso_(H) = 1.2U_eq_(C,N).

### Crystal data

**Table:**

C_7_H_10_N^+^·BF_4_^−^·C_12_H_24_O_6_	*Z* = 2
*M**_r_* = 459.28	*F*(000) = 488
Triclinic, *P*1	*D*_x_ = 1.364 Mg m^−^^3^
Hall symbol: -P 1	Mo *K*α radiation, λ = 0.71073 Å
*a* = 9.281 (6) Å	Cell parameters from 2953 reflections
*b* = 10.673 (6) Å	θ = 2.9–27.5°
*c* = 11.863 (7) Å	µ = 0.12 mm^−^^1^
α = 76.418 (16)°	*T* = 293 K
β = 86.244 (17)°	Prism, colorless
γ = 78.274 (15)°	0.40 × 0.30 × 0.20 mm
*V* = 1118.2 (12) Å^3^	

### Data collection

**Table:**

Rigaku SCXmini diffractometer	5057 independent reflections
Radiation source: fine-focus sealed tube	4153 reflections with *I* > 2σ(*I*)
graphite	*R*_int_ = 0.031
Detector resolution: 28.5714 pixels mm^-1^	θ_max_ = 27.5°, θ_min_ = 2.7°
ω scans	*h* = −12→12
Absorption correction: multi-scan (*CrystalClear*; Rigaku, 2005)	*k* = −13→13
*T*_min_ = 0.958, *T*_max_ = 0.976	*l* = −15→15
12286 measured reflections	

### Refinement

**Table:**

Refinement on *F*^2^	Primary atom site location: structure-invariant direct methods
Least-squares matrix: full	Secondary atom site location: difference Fourier map
*R*[*F*^2^ > 2σ(*F*^2^)] = 0.045	Hydrogen site location: inferred from neighbouring sites
*wR*(*F*^2^) = 0.103	H-atom parameters constrained
*S* = 1.03	*w* = 1/[σ^2^(*F*_o_^2^) + (0.0454*P*)^2^ + 0.2316*P*] where *P* = (*F*_o_^2^ + 2*F*_c_^2^)/3
5057 reflections	(Δ/σ)_max_ < 0.001
280 parameters	Δρ_max_ = 0.25 e Å^−^^3^
0 restraints	Δρ_min_ = −0.24 e Å^−^^3^

### Special details

**Table:**

Geometry. All e.s.d.'s (except the e.s.d. in the dihedral angle between two l.s. planes) are estimated using the full covariance matrix. The cell e.s.d.'s are taken into account individually in the estimation of e.s.d.'s in distances, angles and torsion angles; correlations between e.s.d.'s in cell parameters are only used when they are defined by crystal symmetry. An approximate (isotropic) treatment of cell e.s.d.'s is used for estimating e.s.d.'s involving l.s. planes.
Refinement. Refinement of *F*^2^ against ALL reflections. The weighted *R*-factor *wR* and goodness of fit *S* are based on *F*^2^, conventional *R*-factors *R* are based on *F*, with *F* set to zero for negative *F*^2^. The threshold expression of *F*^2^ > σ(*F*^2^) is used only for calculating *R*-factors(gt) *etc*. and is not relevant to the choice of reflections for refinement. *R*-factors based on *F*^2^ are statistically about twice as large as those based on *F*, and *R*- factors based on ALL data will be even larger.

### Fractional atomic coordinates and isotropic or equivalent isotropic displacement parameters (Å^2^)

**Table:**

	*x*	*y*	*z*	*U*_iso_*/*U*_eq_	
C8	−0.24636 (17)	0.50111 (14)	0.10270 (14)	0.0196 (3)	
H8A	−0.3497	0.5175	0.0841	0.024*	
H8B	−0.1973	0.5562	0.0418	0.024*	
C9	−0.23023 (16)	0.53375 (15)	0.21673 (14)	0.0194 (3)	
H9A	−0.2804	0.6232	0.2153	0.023*	
H9B	−0.2738	0.4751	0.2785	0.023*	
C10	0.27734 (17)	0.10959 (14)	0.00480 (13)	0.0189 (3)	
H10A	0.2860	0.0238	0.0573	0.023*	
H10B	0.3479	0.1025	−0.0585	0.023*	
C11	−0.05246 (17)	0.57472 (15)	0.33132 (13)	0.0203 (3)	
H11A	−0.1023	0.5345	0.4007	0.024*	
H11B	−0.0922	0.6684	0.3133	0.024*	
C12	0.31163 (16)	0.37847 (15)	0.42392 (13)	0.0188 (3)	
H12A	0.3308	0.2915	0.4748	0.023*	
H12B	0.3345	0.4397	0.4657	0.023*	
C13	0.12451 (16)	0.15430 (14)	−0.04151 (12)	0.0176 (3)	
H13A	0.1122	0.2444	−0.0861	0.021*	
H13B	0.1080	0.0996	−0.0923	0.021*	
C14	−0.12985 (16)	0.18795 (15)	0.01623 (13)	0.0186 (3)	
H14A	−0.1928	0.1430	0.0735	0.022*	
H14B	−0.1384	0.1631	−0.0563	0.022*	
C15	−0.18267 (17)	0.33378 (15)	−0.00050 (13)	0.0194 (3)	
H15A	−0.1180	0.3802	−0.0547	0.023*	
H15B	−0.2811	0.3590	−0.0315	0.023*	
C16	0.48137 (16)	0.27382 (15)	0.16325 (13)	0.0180 (3)	
H16A	0.5828	0.2546	0.1875	0.022*	
H16B	0.4647	0.3574	0.1072	0.022*	
C17	0.45350 (16)	0.16760 (15)	0.10879 (13)	0.0189 (3)	
H17A	0.5241	0.1570	0.0461	0.023*	
H17B	0.4648	0.0849	0.1660	0.023*	
C18	0.41154 (16)	0.37904 (14)	0.31845 (13)	0.0184 (3)	
H18A	0.3924	0.4650	0.2660	0.022*	
H18B	0.5136	0.3596	0.3414	0.022*	
C29	0.10945 (17)	0.55084 (14)	0.35178 (13)	0.0195 (3)	
H29A	0.1614	0.5798	0.2798	0.023*	
H29B	0.1286	0.5993	0.4070	0.023*	
O1	0.02026 (11)	0.14543 (10)	0.05354 (8)	0.0168 (2)	
O2	0.30731 (11)	0.20280 (9)	0.06498 (9)	0.0164 (2)	
O3	−0.07640 (11)	0.51969 (10)	0.23695 (8)	0.0160 (2)	
O4	0.15806 (11)	0.41358 (9)	0.39606 (9)	0.0170 (2)	
O5	−0.18295 (11)	0.36644 (9)	0.10949 (9)	0.0171 (2)	
O6	0.38446 (11)	0.28133 (10)	0.26150 (9)	0.0165 (2)	
B1	0.40157 (19)	0.76448 (17)	0.26818 (15)	0.0188 (3)	
F1	0.26267 (12)	0.84348 (11)	0.26112 (10)	0.0428 (3)	
F2	0.42418 (11)	0.70503 (10)	0.17371 (8)	0.0311 (2)	
F3	0.41256 (12)	0.66888 (9)	0.37036 (8)	0.0327 (2)	
F4	0.50826 (12)	0.83934 (11)	0.26526 (10)	0.0401 (3)	
N2	0.08837 (13)	0.24571 (11)	0.25008 (10)	0.0149 (3)	
H2A	0.1771	0.2666	0.2431	0.022*	
H2B	0.0208	0.3151	0.2588	0.022*	
H2C	0.0698	0.2211	0.1867	0.022*	
C1	−0.34030 (17)	0.02500 (15)	0.39415 (13)	0.0193 (3)	
H1A	−0.4325	0.0021	0.4017	0.023*	
C2	−0.23098 (17)	−0.03380 (14)	0.32762 (13)	0.0186 (3)	
H2D	−0.2498	−0.0965	0.2907	0.022*	
C3	−0.09306 (16)	0.00054 (14)	0.31579 (12)	0.0171 (3)	
H3A	−0.0195	−0.0406	0.2722	0.020*	
C4	−0.06450 (16)	0.09625 (14)	0.36893 (12)	0.0158 (3)	
C5	−0.17497 (17)	0.15401 (14)	0.43633 (13)	0.0189 (3)	
H5A	−0.1568	0.2172	0.4729	0.023*	
C6	−0.31203 (17)	0.11841 (15)	0.44960 (13)	0.0202 (3)	
H6A	−0.3847	0.1570	0.4955	0.024*	
C7	0.08405 (16)	0.13519 (14)	0.35362 (13)	0.0186 (3)	
H7A	0.1060	0.1616	0.4225	0.022*	
H7B	0.1589	0.0601	0.3446	0.022*	

### Atomic displacement parameters (Å^2^)

**Table:**

	*U*^11^	*U*^22^	*U*^33^	*U*^12^	*U*^13^	*U*^23^
C8	0.0179 (8)	0.0141 (7)	0.0260 (8)	−0.0017 (6)	−0.0072 (6)	−0.0024 (6)
C9	0.0139 (7)	0.0176 (7)	0.0268 (8)	−0.0023 (6)	−0.0005 (6)	−0.0053 (6)
C10	0.0220 (8)	0.0162 (7)	0.0199 (8)	−0.0036 (6)	0.0026 (6)	−0.0077 (6)
C11	0.0247 (8)	0.0196 (7)	0.0169 (7)	0.0011 (6)	−0.0016 (6)	−0.0092 (6)
C12	0.0185 (8)	0.0198 (7)	0.0192 (7)	−0.0042 (6)	−0.0039 (6)	−0.0047 (6)
C13	0.0231 (8)	0.0161 (7)	0.0149 (7)	−0.0055 (6)	0.0027 (6)	−0.0055 (6)
C14	0.0175 (7)	0.0207 (7)	0.0200 (8)	−0.0065 (6)	−0.0030 (6)	−0.0062 (6)
C15	0.0208 (8)	0.0211 (7)	0.0169 (7)	−0.0050 (6)	−0.0054 (6)	−0.0030 (6)
C16	0.0131 (7)	0.0207 (7)	0.0196 (7)	−0.0041 (6)	0.0031 (6)	−0.0037 (6)
C17	0.0140 (7)	0.0201 (7)	0.0208 (8)	0.0006 (6)	0.0009 (6)	−0.0045 (6)
C18	0.0172 (7)	0.0174 (7)	0.0230 (8)	−0.0060 (6)	−0.0012 (6)	−0.0072 (6)
C29	0.0256 (8)	0.0151 (7)	0.0191 (8)	−0.0035 (6)	−0.0047 (6)	−0.0058 (6)
O1	0.0165 (5)	0.0191 (5)	0.0152 (5)	−0.0044 (4)	0.0002 (4)	−0.0040 (4)
O2	0.0147 (5)	0.0149 (5)	0.0206 (5)	−0.0014 (4)	−0.0010 (4)	−0.0073 (4)
O3	0.0139 (5)	0.0184 (5)	0.0169 (5)	−0.0021 (4)	−0.0010 (4)	−0.0070 (4)
O4	0.0175 (5)	0.0151 (5)	0.0189 (5)	−0.0033 (4)	−0.0019 (4)	−0.0044 (4)
O5	0.0207 (5)	0.0131 (5)	0.0174 (5)	−0.0020 (4)	−0.0043 (4)	−0.0030 (4)
O6	0.0157 (5)	0.0172 (5)	0.0196 (5)	−0.0063 (4)	0.0032 (4)	−0.0080 (4)
B1	0.0194 (9)	0.0190 (8)	0.0189 (8)	−0.0056 (7)	−0.0011 (7)	−0.0040 (7)
F1	0.0308 (6)	0.0468 (7)	0.0417 (7)	0.0136 (5)	−0.0010 (5)	−0.0099 (5)
F2	0.0356 (6)	0.0352 (6)	0.0263 (5)	−0.0055 (4)	−0.0027 (4)	−0.0151 (4)
F3	0.0475 (6)	0.0251 (5)	0.0250 (5)	−0.0125 (5)	−0.0064 (5)	0.0013 (4)
F4	0.0464 (7)	0.0388 (6)	0.0443 (7)	−0.0292 (5)	−0.0010 (5)	−0.0094 (5)
N2	0.0148 (6)	0.0156 (6)	0.0149 (6)	−0.0038 (5)	0.0005 (5)	−0.0040 (5)
C1	0.0156 (7)	0.0234 (8)	0.0170 (7)	−0.0051 (6)	−0.0007 (6)	0.0005 (6)
C2	0.0220 (8)	0.0183 (7)	0.0170 (7)	−0.0072 (6)	−0.0020 (6)	−0.0037 (6)
C3	0.0182 (7)	0.0181 (7)	0.0141 (7)	−0.0026 (6)	0.0020 (6)	−0.0032 (6)
C4	0.0179 (7)	0.0152 (7)	0.0127 (7)	−0.0053 (6)	−0.0033 (6)	0.0026 (5)
C5	0.0249 (8)	0.0156 (7)	0.0160 (7)	−0.0037 (6)	−0.0026 (6)	−0.0026 (6)
C6	0.0194 (8)	0.0206 (8)	0.0176 (7)	0.0001 (6)	0.0021 (6)	−0.0028 (6)
C7	0.0180 (8)	0.0174 (7)	0.0185 (7)	−0.0054 (6)	−0.0042 (6)	0.0024 (6)

### Geometric parameters (Å, °)

**Table:**

C8—O5	1.4236 (19)	C16—H16B	0.9700
C8—C9	1.497 (2)	C17—O2	1.4303 (19)
C8—H8A	0.9700	C17—H17A	0.9700
C8—H8B	0.9700	C17—H17B	0.9700
C9—O3	1.4351 (19)	C18—O6	1.4376 (18)
C9—H9A	0.9700	C18—H18A	0.9700
C9—H9B	0.9700	C18—H18B	0.9700
C10—O2	1.4313 (18)	C29—O4	1.4235 (19)
C10—C13	1.498 (2)	C29—H29A	0.9700
C10—H10A	0.9700	C29—H29B	0.9700
C10—H10B	0.9700	B1—F3	1.383 (2)
C11—O3	1.4290 (18)	B1—F1	1.385 (2)
C11—C29	1.498 (2)	B1—F4	1.387 (2)
C11—H11A	0.9700	B1—F2	1.397 (2)
C11—H11B	0.9700	N2—C7	1.4946 (19)
C12—O4	1.4380 (19)	N2—H2A	0.8900
C12—C18	1.507 (2)	N2—H2B	0.8900
C12—H12A	0.9700	N2—H2C	0.8900
C12—H12B	0.9700	C1—C2	1.384 (2)
C13—O1	1.4355 (18)	C1—C6	1.390 (2)
C13—H13A	0.9700	C1—H1A	0.9300
C13—H13B	0.9700	C2—C3	1.391 (2)
C14—O1	1.4395 (19)	C2—H2D	0.9300
C14—C15	1.503 (2)	C3—C4	1.395 (2)
C14—H14A	0.9700	C3—H3A	0.9300
C14—H14B	0.9700	C4—C5	1.392 (2)
C15—O5	1.4272 (19)	C4—C7	1.507 (2)
C15—H15A	0.9700	C5—C6	1.389 (2)
C15—H15B	0.9700	C5—H5A	0.9300
C16—O6	1.4322 (18)	C6—H6A	0.9300
C16—C17	1.501 (2)	C7—H7A	0.9700
C16—H16A	0.9700	C7—H7B	0.9700
			
O5—C8—C9	109.25 (12)	O2—C17—H17B	109.9
O5—C8—H8A	109.8	C16—C17—H17B	109.9
C9—C8—H8A	109.8	H17A—C17—H17B	108.3
O5—C8—H8B	109.8	O6—C18—C12	108.68 (12)
C9—C8—H8B	109.8	O6—C18—H18A	110.0
H8A—C8—H8B	108.3	C12—C18—H18A	110.0
O3—C9—C8	108.54 (12)	O6—C18—H18B	110.0
O3—C9—H9A	110.0	C12—C18—H18B	110.0
C8—C9—H9A	110.0	H18A—C18—H18B	108.3
O3—C9—H9B	110.0	O4—C29—C11	107.80 (12)
C8—C9—H9B	110.0	O4—C29—H29A	110.1
H9A—C9—H9B	108.4	C11—C29—H29A	110.1
O2—C10—C13	108.95 (12)	O4—C29—H29B	110.1
O2—C10—H10A	109.9	C11—C29—H29B	110.1
C13—C10—H10A	109.9	H29A—C29—H29B	108.5
O2—C10—H10B	109.9	C13—O1—C14	112.74 (12)
C13—C10—H10B	109.9	C17—O2—C10	111.77 (11)
H10A—C10—H10B	108.3	C11—O3—C9	111.85 (11)
O3—C11—C29	109.20 (12)	C29—O4—C12	113.18 (11)
O3—C11—H11A	109.8	C8—O5—C15	111.32 (11)
C29—C11—H11A	109.8	C16—O6—C18	110.94 (11)
O3—C11—H11B	109.8	F3—B1—F1	109.87 (14)
C29—C11—H11B	109.8	F3—B1—F4	109.19 (13)
H11A—C11—H11B	108.3	F1—B1—F4	110.24 (14)
O4—C12—C18	113.11 (12)	F3—B1—F2	109.64 (14)
O4—C12—H12A	109.0	F1—B1—F2	108.85 (13)
C18—C12—H12A	109.0	F4—B1—F2	109.04 (14)
O4—C12—H12B	109.0	C7—N2—H2A	109.5
C18—C12—H12B	109.0	C7—N2—H2B	109.5
H12A—C12—H12B	107.8	H2A—N2—H2B	109.5
O1—C13—C10	109.27 (13)	C7—N2—H2C	109.5
O1—C13—H13A	109.8	H2A—N2—H2C	109.5
C10—C13—H13A	109.8	H2B—N2—H2C	109.5
O1—C13—H13B	109.8	C2—C1—C6	119.87 (14)
C10—C13—H13B	109.8	C2—C1—H1A	120.1
H13A—C13—H13B	108.3	C6—C1—H1A	120.1
O1—C14—C15	112.86 (12)	C1—C2—C3	120.23 (14)
O1—C14—H14A	109.0	C1—C2—H2D	119.9
C15—C14—H14A	109.0	C3—C2—H2D	119.9
O1—C14—H14B	109.0	C2—C3—C4	120.39 (14)
C15—C14—H14B	109.0	C2—C3—H3A	119.8
H14A—C14—H14B	107.8	C4—C3—H3A	119.8
O5—C15—C14	108.15 (12)	C5—C4—C3	118.85 (14)
O5—C15—H15A	110.1	C5—C4—C7	120.99 (14)
C14—C15—H15A	110.1	C3—C4—C7	120.15 (13)
O5—C15—H15B	110.1	C6—C5—C4	120.80 (14)
C14—C15—H15B	110.1	C6—C5—H5A	119.6
H15A—C15—H15B	108.4	C4—C5—H5A	119.6
O6—C16—C17	109.34 (12)	C5—C6—C1	119.85 (14)
O6—C16—H16A	109.8	C5—C6—H6A	120.1
C17—C16—H16A	109.8	C1—C6—H6A	120.1
O6—C16—H16B	109.8	N2—C7—C4	111.37 (12)
C17—C16—H16B	109.8	N2—C7—H7A	109.4
H16A—C16—H16B	108.3	C4—C7—H7A	109.4
O2—C17—C16	109.06 (12)	N2—C7—H7B	109.4
O2—C17—H17A	109.9	C4—C7—H7B	109.4
C16—C17—H17A	109.9	H7A—C7—H7B	108.0
			
O5—C8—C9—O3	64.50 (15)	C9—C8—O5—C15	−174.28 (12)
O2—C10—C13—O1	−68.12 (15)	C14—C15—O5—C8	−173.89 (12)
O1—C14—C15—O5	−64.33 (16)	C17—C16—O6—C18	177.97 (12)
O6—C16—C17—O2	64.18 (15)	C12—C18—O6—C16	−177.47 (12)
O4—C12—C18—O6	−62.79 (15)	C6—C1—C2—C3	−0.2 (2)
O3—C11—C29—O4	−68.82 (15)	C1—C2—C3—C4	−1.2 (2)
C10—C13—O1—C14	179.48 (11)	C2—C3—C4—C5	1.7 (2)
C15—C14—O1—C13	−86.11 (15)	C2—C3—C4—C7	−178.64 (13)
C16—C17—O2—C10	176.00 (12)	C3—C4—C5—C6	−0.7 (2)
C13—C10—O2—C17	−178.41 (12)	C7—C4—C5—C6	179.59 (13)
C29—C11—O3—C9	177.14 (12)	C4—C5—C6—C1	−0.7 (2)
C8—C9—O3—C11	167.91 (12)	C2—C1—C6—C5	1.1 (2)
C11—C29—O4—C12	−177.59 (11)	C5—C4—C7—N2	−90.62 (17)
C18—C12—O4—C29	−76.33 (15)	C3—C4—C7—N2	89.72 (17)

### Hydrogen-bond geometry (Å, °)

**Table:**

*D*—H···*A*	*D*—H	H···*A*	*D*···*A*	*D*—H···*A*
N2—H2A···O6	0.89	1.99	2.866 (2)	167
N2—H2B···O3	0.89	2.15	2.986 (2)	157
N2—H2C···O1	0.89	2.05	2.936 (2)	173

## References

[bb1] Akutagawa, T., Hashimoto, A., Nishihara, S., Hasegawa, T. & Nakamura, T. (2002). *J. Supramol. Chem.* **2**, 175–186.

[bb2] Ferguson, G. (1999). *PRPKAPPA* University of Guelph, Canada.

[bb3] Kryatova, O. P., Korendovych, I. V. & Rybak-Akimova, E. V. (2004). *Tetrahedron*, **60**, 4579–4588.

[bb4] Rigaku (2005). *CrystalClear* Rigaku Corporation, Tokyo, Japan.

[bb5] Sheldrick, G. M. (2008). *Acta Cryst.* A**64**, 112–122. 10.1107/S0108767307043930 18156677

[bb6] Ye, H. Y., Fu, D. W., Zhang, Y., Zhang, W., Xiong, R. G. & Huang, S. P. (2009). *J. Am. Chem. Soc.* **131**, 42–43. 10.1021/ja808331g 19128170

[bb7] Zhang, W., Cheng, L. Z., Xiong, R. G., Nakamura, T. & Huang, S. P. (2009). *J. Am. Chem. Soc.* **131**, 12544–12545. 10.1021/ja905399x 19685869

